# Phosphodiesterases 2, 3 and 4 can decrease cardiac effects of H_2_-histamine-receptor activation in isolated atria of transgenic mice

**DOI:** 10.1007/s00210-021-02052-y

**Published:** 2021-02-12

**Authors:** Joachim Neumann, Rafaela Voss, Ulrich Laufs, Christian Werner, Ulrich Gergs

**Affiliations:** 1 Institut für Pharmakologie und Toxikologie, Medizinische Fakultät, Martin-Luther-Universität Halle-Wittenberg, Magdeburger Str. 4, D-06112 Halle, Germany; 2 Klinik und Poliklinik für Kardiologie, Universitätsklinikum Leipzig, Liebigstraße 20, 04103 Leipzig, Germany; 3 Klinik für Innere Medizin III -Kardiologie, Angiologie und Internistische Intensivmedizin, Universitätsklinikum des Saarlandes, Kirrberger Straße, Geb. 41.1, D-66421 Homburg, Saar Germany

**Keywords:** Histamine, Inotropy, Chronotropy, Transgenic mice, Phosphodiesterase, H_2_-histamine receptor

## Abstract

Histamine exerts cAMP-dependent positive inotropic effects (PIE) and positive chronotropic effects (PCE) on isolated left and right atria, respectively, of transgenic mice which overexpress the human H_2_-receptor in the heart (=H_2_-TG). To determine whether these effects are antagonized by phosphodiesterases (PDEs), contractile studies were done in isolated left and right atrial preparations of H_2_-TG. The contractile effects of histamine were tested in the additional presence of the PDE-inhibitors*erythro*-9-(2-hydroxy-3-nonyl)adenine hydrochloride (EHNA, 1 μM, PDE2-inhibitor) or cilostamide (1 μM, PDE3-inhibitor), rolipram (10 μM, a PDE4-inhibitor), and their combinations. Cilostamide (1 μM) and EHNA (1 μM), rolipram (1 μM), and EHNA (1 μM) and the combination of rolipram (0.1 μM) and cilostamide (1 μM) each increased the potency of histamine to elevate the force of contraction (FOC) in H_2_-TG. Cilostamide (1 μM) and rolipram (10 μM) alone increased and EHNA (1 μM) decreased alone, and their combination increased the potency of histamine to increase the FOC in H_2_-TG indicating that PDE3 and PDE4 regulate the inotropic effects of histamine in H_2_-TG. The PDE inhibitors (EHNA, cilostamide, rolipram) alone did not alter the potency of histamine to increase the heart beat in H_2_-TG whereas a combination of rolipram, cilostamide, and EHNA, or of rolipram and EHNA increased the potency of histamine to act on the beating rate. In summary, the data suggest that the PCE of histamine in H_2_-TG atrium involves PDE 2 and 4 activities, whereas the PIE of histamine are diminished by activity of PDE 3 and 4.

## Introduction

The effects of histamine are mediated by receptors known as H_1_, H_2_, H_3_, and H_4_-histamine receptors (Jutel et al., 2009). The cardiac H_2_-histamine-receptors mediate the PIE of histamine in isolated human cardiac preparations (Levi et al., 1981). These PIE in the human heart were accompanied by and hence probably mediated by an increase in cAMP (atrial preparations: Sanders et al., 1996). We failed to notice a PIE or PCE of histamine in WT atrium of mice (Gergs et al., 2019b, 2020) and therefore, we generated mice overexpressing human H_2_ receptors and in those mice detected an increase in force of contraction and the beating rate by histamine (Gergs et al., 2019b, 2020, this manuscript).

Histamine acts in many ways similar to serotonin in the heart. Indeed, in the human failing ventricle, treatment of trabeculae carneae with 3-isobutyl-1-methylxanthine (IBMX), an unspecific phosphodiesterase (PDE) inhibitor, uncovered a hidden effect: under these conditions, 5-HT elicited a PIE via 5-HT_4_-receptor receptors (Brattelid et al., 2004). The same group reiterated some of these effects in a rat model. Rat ventricular preparations express both 5-HT_2A_ and 5-HT_4_ receptors on the mRNA level but signal only through 5-HT_2A_(Brattelid et al., 2012, Läer et al., 1998) under normal conditions. However, with aortic banding to induce hypertrophy in rats, the authors noticed that a PIE of 5-HT via 5-HT_4_ receptors could be detected.

The degradation of cAMP is solely brought about by cAMP-specific PDEs. PDEs degrade cAMP to inactive 5´-AMP and modulate cAMP-dependent signaling. Thus, PDEs in the heart will diminish or even terminate the responses to receptors, such as β-adrenoceptors, 5-HT_4_ receptors, or H_2_-receptors, that lead to cAMP production.

Among these PDEs are PDE1, a Ca^2+^/calmodulin-activated PDE; PDE2, a cGMP-activated PDE; PDE3, a cGMP-inhibited PDE; and PDE4, a cGMP-independent, cAMP-specific PDE. PDEs can be classified as cAMP selective (PDE4, 7, 8) or cGMP selective (PDE5, 6, 9), or hydrolyzing both cAMP and cGMP (PDE1, 2, 3, 10, 11) (Bobin et al., 2016, Conti and Beavo, 2007). PDE2, PDE3, and PDE4 provide the major PDE activity for cAMP in the heart. To dissect the role of PDEs for inotropy and chronotropy in the heart, drugs that are specific inhibitors have been used in many previous studies. This study used the same inhibitors as previous investigators at the same concentrations to facilitate interpretation of the present work. Specifically, this study used *erythro*-9-(2-Hydroxy-3-nonyl)adenine hydrochloride (EHNA), as a PDE2-inhibitor; cilostamide, as a PDE3-inhibitor; and rolipram, as a PDE4-inhibitor(for specificity, see Maurice et al., 2014, Table 1 in Gergs et al., 2019b).

**Table 1:** Contractile time parameters (time to peak tension= t1, time of relaxation =t2 in left atrial preparations in the absence or presence of PDE inhibitors and the combinations thereof. Numbers of experiments in each group was four to seven

t1	TG Ctr 1	TG Ctr 2	TG His 1 μM	WT Ctr 1	WT Ctr 2	WT His 1 μM
Without PDE-Inhibitor	13.2 ± 0.1	-	12.2 ± 0.1*	13.4 ± 0.1	-	13.6 ± 0.1^+^
EHNA	12.9 ± 0.2	12.9 ± 0.1	12.5 ± 0.3	12.8 ± 0.2	12.9 ± 0.2	13.0 ± 0.2
Cilostamide	12.6 ± 0.2	12.7 ± 0.2	11.4 ± 0.1^#^	12.9 ± 0.1	12.9 ± 0.1	13.1 ± 0.2^+^
Rolipram 10 μM	13.0 ± 0.2	12.7 ± 0.2	11.5 ± 0.2^#^	12.3 ± 0.2	12.1 ± 0.2	12.0 ± 0.2
Cilo + EHNA	14.2 ± 0.3	14.1 ± 0.3	12.4 ± 0.2^#^	14.8 ± 0.2	14.5 ± 0.2	14.6 ± 0.2^+^
Roli 10 μM + Cilo	14.4 ± 0.2	12.9 ± 0.2*	12.6 ± 0.2*	14.9 ± 0.3	12.7 ± 0.2*	12.4 ± 0.1
Roli 1 μM + Cilo	14.2 ± 0.2	12.7 ± 0.1*	12.3 ± 0.1*	14.5 ± 0.2	12.7 ± 0.1*	12.4 ± 0.1
Roli 0.1 μM + Cilo	13.9 ± 0.3	13.2 ± 0.3*	12.5 ± 0.1	14.9 ± 0.7	13.1 ± 0.2*	13.1 ± 0.3
Roli 1 μM + EHNA	14.0 ± 0.2	13.6 ± 0.3*	12.6 ± 0.1^#^	14.7 ± 0.2	14.2 ± 0.2	14.1 ± 0.3^+^
Roli 0.1 μM + Cilo + EHNA	14.0 ± 0.2	14.4 ± 0.7	12.4 ± 0.2	14.3 ± 0.2	13.1 ± 0.1*	12.9 ± 0.1
**t2**	**TG Ctr 1**	**TG Ctr 2**	**TG His 1 μM**	**WT Ctr 1**	**WT Ctr 2**	**WT His 1 μM**
Without PDE-Inhibitor	32.3 ± 0.8	-	27.7 ± 0.4*	33.6 ± 0.7	-	33.4 ± 0.7^+^
EHNA	32.9 ± 2.5	32.7 ± 2.4	27.6 ± 0.9	34.3 ± 3.3	34.1 ± 3.2	33.9 ± 3.2
Cilostamide	27.9 ± 1.2	28.4 ± 1.3	25.8 ± 0.8	38.0 ± 2.6^+^	34.7 ± 1.9	33.2 ± 2.2
Rolipram 10 μM	30.7 ± 2.5	28.9 ± 2.7	26.1 ± 0.7	27.1 ± 1.4	26.1 ± 0.9	24.5 ± 0.9
Cilo + EHNA	29.2 ± 1.0	27.9 ± 0.7	26.4 ± 0.6	30.1 ± 0.9	28.0 ± 0.7	27.2 ± 0.8
Roli 10 μM + Cilo	24.4 ± 1.0	24.1 ± 1.2	24.4 ± 0.9	31.5 ± 2.1^+^	28.5 ± 1.0	27.0 ± 0.9
Roli 1 μM + Cilo	24.9 ± 1.9	26.6 ± 1.3	26.3 ± 1.1	30.68 ± 2.1	29.0 ± 0.8	27.1 ± 0.7
Roli 0.1 μM + Cilo	23.3 ± 1.1	23.6 ± 0.9	26.1 ± 0.6^#^	25.8 ± 1.9	25.8 ± 1.4	23.9 ± 0.9
Roli 1 μM + EHNA	21.5 ± 1.1	20.9 ± 1.0	25.3 ± 0.5^#^	29.1 ± 2.3	27.1 ± 2.3	25.0 ± 1.7
Roli 0.1 μM + Cilo + EHNA	23.7 ± 1.5	21.7 ± 1.0	24.8 ± 1.1^#^	24.8 ± 2.0	22.7 ± 1.8	21.2 ± 1.6

*p < 0.05 vs. Ctr1; ^#^*p* < 0.05 vs. Ctr2; ^+^*p* < 0.05 vs. TG

The present study was started to determine whether the inotropic and chronotropic effects of histamine in our mouse model, that mimics the cardiac effects of histamine on PIE and PCE by H_2_-histamine receptors (H_2_-TG), are sensitive to typical PDE inhibitors in the atrium of transgenic mice engineered to express a functional H_2_-receptor on atrial and ventricular cardiomyocytes (Gergs et al., 2019a). This study tested whether or not PDE 2, 3, or 4 alone or in concert are important for H_2_-receptor-mediated effects in the contracting left atrium (electrically driven) and in the sinus node of the spontaneously beating right atrium of H_2_-TG.

## Materials and methods

### Transgenic mice

Transgenic mice (H_2_-TG) with cardiac myocyte-specific overexpression of the human H_2_- histamine receptor and their littermate control mice (WT) were generated as described by Gergs et al. (2019a). Heart-specific expression was achieved via the α-myosin heavy-chain promoter. The animals were in average about 150 days of age (75 female and 65 male animals). Contraction experiments were performed on left and right atrial preparations as previously described by Gergs et al. (2013). All mice were housed under conditions of optimum light, temperature, and humidity with food and water provided ad libitum. Animals were handled and maintained according to approved protocols of the animal welfare committee of the University of Halle-Wittenberg, Halle, Germany, (approval reference number 42502-02-691 MLU).

### Contractile studies in mice

In brief, right or left atrial preparations were isolated and mounted in organ baths as described by Gergs et al. (2013, 2017, 2019b) and Neumann et al. (2003). The organ baths’ bathing solution contained 119.8 mM NaCl 5.4 mM KCl, 1.8 mM CaCl_2_, 1.05 mM MgCl_2_, 0.42 mM NaH_2_PO_4_, 22.6 mM NaHCO_3_, 0.05 mM Na_2_EDTA, 0.28 mM ascorbic acid, and 5.05 mM glucose. It was continuously gassed with 95% O_2_ and 5% CO_2_ and maintained at 37 °C and pH 7.4, as described by Neumann et al. (2003) and Kirchhefer et al. (2004). Preparations were attached to a bipolar stimulating electrode and suspended individually in 10-ml glass tissue chambers for recording isometric contractions. Force of contraction (FOC) was measured with inductive force transducers connected to a chart recorder. Time parameters (time to peak tension =t1 or time of tension relaxation =t2 in milli seconds, ms) and minimum and maximum of the first derivate versus time of force of contraction with respect to time of single contractions (dF/dT_min_ and dF/dT_max_ in milli Newton per milli second, mN/ms) were evaluated from digitized recordings. Each muscle was stretched to the length of maximal FOC. The left atrial preparations from mice were electrically stimulated at 1 Hz with rectangular pulses of 5 ms duration; the voltage was ~ 10–20% greater than the threshold of initiation of contraction. Spontaneously beating right atrial preparations from mice were used to study any chronotropic effects.

### Banding

Aortic constriction of mice was performed as described previously (Müller et al., 2009).

### Data analysis

Data shown are means ± standard error of the mean. Statistical significance was estimated by analysis of variance followed by Bonferroni´s *t* test. A *p* value of less than 0.05 was considered significant. Experimental data for agonist-induced positive inotropic and chronotropic effects were analyzed by fitting sigmoidal curves to the experimental data with GraphPad Prism 5.0. All other statistical analyses were performed as indicated in the figures and tables. Statistical evaluation was conducted with GraphPad Prism 5.0 (GraphPad Software, San Diego, California, USA).

### Drugs and materials

(-)-Isoprenaline (+)-bitartrate, serotonin (5-HT) hydrochloride, and histamine were purchased from Sigma-Aldrich (Deisenhofen, Germany). Rolipram, EHNA, and cilostamide were obtained from Tocris (Wiesbaden, Germany). All other chemicals were of the highest purity grade commercially available. Deionized water was used throughout the experiments. Stock solutions were freshly prepared daily.

## Results

To facilitate comparisons, all EC-50 values have been put together in Table 3.

### Effects without PDE inhibitors

**Fig. 1:**
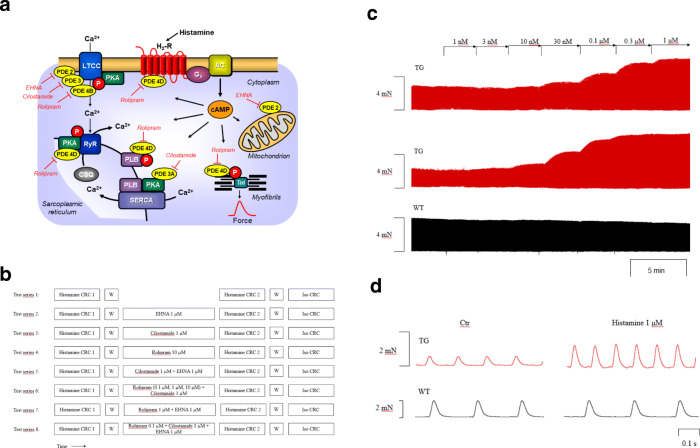
**a** Scheme of the putative subcellular localization of phosphodiesterase (PDEs) 2-4. Their inhibitors used in this study are also depicted. Ca^2+^ enters the mammalian heart cell via the L-type Ca^2+^ channel (LTCC). This process can be enhanced by histamine via a cascade starting with the H_2_-receptor, the occupation of which by histamine elevates the activity of adenylyl cyclase (AC) in the sarcolemma via stimulatory G-proteins (G_s_), elevates subsequent production of cAMP and, thereby, activates cAMP-dependent protein kinase (PKA). PKA increases cardiac force generation and relaxation by increasing the phosphorylation state (P) of LTCC, phospholamban (PLB), and other regulatory proteins. Trigger Ca^2+^ initiates release of Ca^2+^ from the sarcoplasmic reticulum via ryanodine receptors (RYR) into the cytosol, where Ca^2+^ activates myofilaments and leads to increased inotropy. In diastole, Ca^2+^ is taken up into the sarcoplasmic reticulum via a sarcoplasmic reticulum Ca^2+^ ATPase (SERCA), whose activity is higher when the phosphorylation state of PLB is elevated by PKA. PDE2-4 have been localized to the LTCC, PDE3 and PDE4 to PLB, PDE4 to G-protein coupled receptors, RYR, and the myofilaments, and PDE2 to the cytosol. Not shown here: PDE3 and PDE4 are also localized to the nucleus. EHNA preferentially inhibits PDE2, cilostamide PDE3, rolipram PDE4. **b** Schematic description of the order of drug application to isolated atria, including incubation times and concentrations of drugs. **c** Original recordings of the force of contraction (FOC) in left atria from transgenic mice that overexpress the H_2_-receptor (H_2_-TG) (top), in the presence of 0.1 μM rolipram,cilostamide and EHNA (H_2_-TG, middle tracing) and littermate control (WT) (bottom). Concentration response curves for histamine are shown. Note the fast inset of action and the concentration dependence of the positive inotropic effect (PIE) in H_2_-TG (top), the leftward shift of the curve (middle) and the lack of any PIE in WT (bottom). **d** Original recordings of the FOC in right atria from H_2_-TG (top) and WT (bottom). High temporal resolution is shown (see time bar) to make two single contractions visible. Note that the time between beats is shortened (positive chronotropic effect = PCE) of 1 μM histamine in H_2_-TG and the lack of a PIE in WT

**Fig. 2:**
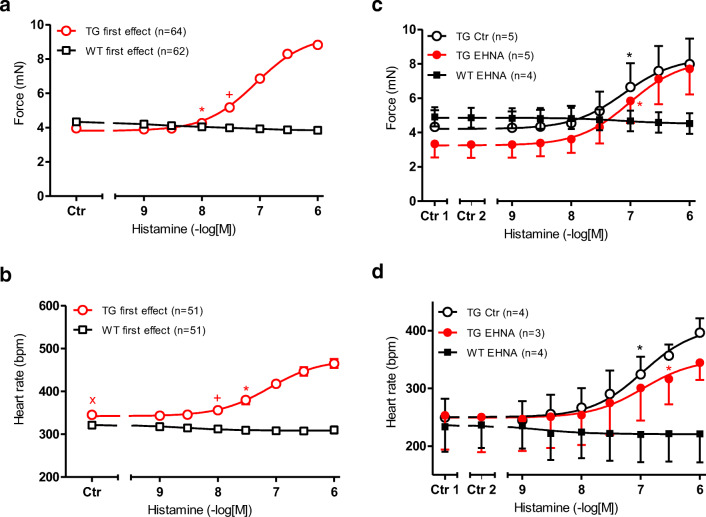
**a** Effect of histamine alone on the FOC of isolated electrically driven (1 Hz) left atrium of H_2_-TG (closed circles) or WT (squares). FOC before histamine is indicated as Ctr (control). First effect in the figure means that all left atrial contraction experiments with the initial concentration response curve to histamine (please compare Fig. 1b for the experimental design) in all groups #1-#8 are summarized here. Numbers in brackets mean number of experiments. ^★, **+**^indicate first significant difference (*p* < 0.05) vs. Ctr or WT. **b** Effects of histamine alone in isolated spontaneously beating right atrium of H_2_-TG (closed circles) or WT (squares). FOC before histamine addition is indicated as Ctr. Beating rate in beats per minute (bpm) is depicted on the ordinate. First effect in the figure means that all right atrial contraction experiments with the initial concentration response curve to histamine (please compare Fig. 1b for the experimental design) in all groups #1-#8 are summarized here. Numbers in brackets indicate number of experiments. ^★, **+**^indicate first significant difference (*p* < 0.05) vs. Ctr or WT, x indicates a significantly higher (*p* < 0.05) basal beating rate (in the absence of external histamine) vs WT. **c** Effect of histamine alone (open squares) or in the additional presence of 1 μM *erythro*-9-(2-hydroxy-3-nonyl)adenine hydrochloride (EHNA) on the FOC of isolated electrically driven (1 Hz) left atrium of H_2_-TG (closed circles) or WT (squares). FOC before application of EHNA (=Ctr 1) and after EHNA (=Ctr 2) are indicated. **d** Effect of histamine alone (open squares) alone or in the additional presence of 1 μM EHNA in isolated spontaneously beating right atrium of H_2_-TG (closed circles) or WT (squares). FOC before (Ctr 1) and after EHNA (Ctr 2) are indicated. Beating rate in beats per minute (bpm) is depicted on the ordinate. Abscissae indicate negative decadic logarithm of increasing histamine concentrations. Numbers in brackets indicate number of experiments. ^★, **+**^indicate first significant difference (*p* < 0.05) vs. Ctr 2 or WT

As seen in the original recording (Fig. 1c, top) and summarized in Fig. 2 a (open circles), histamine exerted a PIE in isolated electrically stimulated (1 Hz) left atrial preparations of H_2_-TG that was concentration dependent (-log EC_50_ = − 7.07 ± 0.04 M (*n* = 63), EC_50_: effective concentration in M for 50 % effect) but histamine was completely lacking a PIE in WT (original tracing in Fig. 1c and summarized in Fig. 2a (open squares) in agreement with our published data (Gergs et al. 2019b). At the same time, t_2_ (=time of relaxation) amounted to 33.6 ± 0.74 ms and (*n* = 65) in WT and 32.3 ± 0.81 ms (*n* = 67) in H_2_-TG under basal conditions, and by 1 μM histamine remained unaltered at 33.39 ± 0.74 ms in WT and shortened to 27.71 ± 0.37 ms (*n* = 67) in H_2_-TG (compare Table 1, bottom). In a similar way, 1 μM histamine shortened t_1_ (time to peak tension) in H_2_-TG, but not in WT (see Table 1, top)

Likewise, dF/dT_max_ (=maximum rate of tension development) amounted to 302.6 ± 14.31 mN/ms (*n* = 67) in WT and 271.9 ± 11.80 mN/ms (*n* = 68) in H_2_-TG under basal conditions, and were augmented in H_2_-TG but not WT by 1 μM histamine namely amounted to 267.6 ± 12.82 mN/ms (*n* = 67) in WT and 647.9 ± 18.96 mN/ms (*n* = 68) in H2-TG(compare also Table 2 top). Similarly, 1 μM histamine shortened dF/dT_min_ (=minimum rate of tension development= rate of relaxation) in H_2_-TG but not WT (see Table 2, bottom).

**Table 2:** First derivative of force versus time. Maximum derivative: dF/dT_max_, minimum derivative: dF/dT_min_, in left atrial preparations in the absence or presence of PDE inhibitors and the combinations thereof. Numbers of experiments in each group were four to seven

**dF/dTmax**	**TG Ctr 1**	**TG Ctr 2**	**TG His 1 μM**	**WT Ctr 1**	**WT Ctr 2**	**WT His 1 μM**
Without PDE-Inhibitor	272 ± 12	–	648 ± 19*	303 ± 14	–	268 ± 13^+^
EHNA	281 ± 53	275 ± 51	672 ± 106^#^	353 ± 41	348 ± 42	322 ± 43^+^
Cilostamide	163 ± 37	163 ± 39	647 ± 57^#^	168 ± 41	168 ± 42	118 ± 20^+^
Rolipram 10 μM	202 ± 23	258 ± 35	707 ± 27^#^	195 ± 74	251 ± 64	246 ± 73^+^
Cilo + EHNA	161 ± 24	148 ± 21	613 ± 68^#^	244 ± 32	246 ± 26	215 ± 22^+^
Roli 10 μM + Cilo	157 ± 18	507 ± 90*	600 ± 73	279 ± 45	848 ± 53*	838 ± 50
Roli 1 μM + Cilo	194 ± 39	663 ± 85*	700 ± 83	260 ± 43	710 ± 71*	703 ± 69
Roli 0.1 μM + Cilo	195 ± 27	445 ± 91*	705 ± 48^#^	210 ± 39	514 ± 121	479 ± 126
Roli 1 μM + EHNA	116 ± 12	148 ± 28	608 ± 38^#^	185 ± 38	253 ± 42	235 ± 38
Roli 0.1 μM + Cilo + EHNA	128 ± 36	212 ± 65	603 ± 52^#^	169 ± 24	392 ± 106	338 ± 99
**dF/dTmin**	**TG Ctr 1**	**TG Ctr 2**	**TG His 1 μM**	**WT Ctr 1**	**WT Ctr 2**	**WT His 1 μM**
Without PDE-Inhibitor	− 148 ± 7	–	− 352 ± 11*	− 161 ± 8	–	− 144 ± 7^+^
EHNA	− 198 ± 22	− 194 ± 20	− 465 ± 83^#^	− 189 ± 30	− 188 ± 30	− 173 ± 28^+^
Cilostamide	− 93 ± 17	− 93 ± 18	− 414 ± 48^#^	− 92 ± 21	− 93 ± 21	− 72 ± 15^+^
Rolipram 10 μM	− 123 ± 14	− 163 ± 23	− 420 ± 22^#^	− 109 ± 39	− 162 ± 37	− 181 ± 44^+^
Cilo + EHNA	− 91 ± 12	− 84 ± 11	− 305 ± 26^#^	− 134 ± 16	− 138 ± 13	− 123 ± 11^+^
Roli 10 μM + Cilo	− 94 ± 9	− 272 ± 41*	− 318 ± 35	− 155 ± 20	− 420 ± 27*	− 415 ± 24
Roli 1 μM + Cilo	− 116 ± 22	− 340 ± 40*	− 348 ± 37	− 137 ± 19	− 346 ± 34*	− 347 ± 37
Roli 0.1 μM + Cilo	− 121 ± 16	− 257 ± 47*	− 355 ± 23	− 123 ± 20	− 269 ± 58	− 258 ± 58
Roli 1 μM + EHNA	− 76 ± 7	− 97 ± 17	− 307 ± 16^#^	− 105 ± 19	− 144 ± 22	− 141 ± 22
Roli 0.1 μM + Cilo + EHNA	− 80.8 ± 18	132 ± 32	− 307 ± 20^#^	− 101 ± 10	− 219 ± 46	− 200 ± 47

*p < 0.05 vs. Ctr1; ^#^*p* < 0.05 vs. Ctr2; ^+^*p* < 0.05 vs. TG

Similarly, histamine exerted a concentration-dependent positive chronotropic effect (=PCE, -log EC_50_ = 7.13 ± 0.06, *n* = 43) in right atrial preparations of H_2_-TG (original recording, Fig. 1d, top, summarized in Fig. 2b, (open circles)) but histamine was devoid of any PCE in WT (original tracing: Fig. 1d bottom, Fig. 2b (squares)). In Fig. 2 a and Fig. 2 b, the contractile initial (=first) effects of histamine in all subgroups (Fig. 1b: #1-#8) of studied atria were summarized, which explains the larger number of experiments and small error bars in these figures.

### Effects of PDE inhibitors alone

EHNA (1 μM), a PDE2 inhibitor, alone, was ineffective to increase the FOC in left atrial preparations (Ctr 2 = contractile value after complete stabilization of the effect of a PDE-inhibitor vs. Ctr 1 = pre-drug contractile value) of WT (squares) and H_2_-TG (closed circles) in Fig. 2 c. The effects in the absence of any PDE inhibitor are additionally plotted (open circles, Fig. 2c). Likewise, 1 μM EHNA alone did not increase the beating rate in right atrial preparations from WT or H_2_-TG (Ctr 2 vs. Ctr 1) in Fig. 2 d. EHNA shifted the PIE of histamine in H_2_-TG slightly, but significantly, to the right (circles, -log EC_50_ Ctr = 7.11 ± 0.11; -log EC_50_ EHNA = 7.01 ± 0.14; *p* < 0.05).

**Fig. 3:**
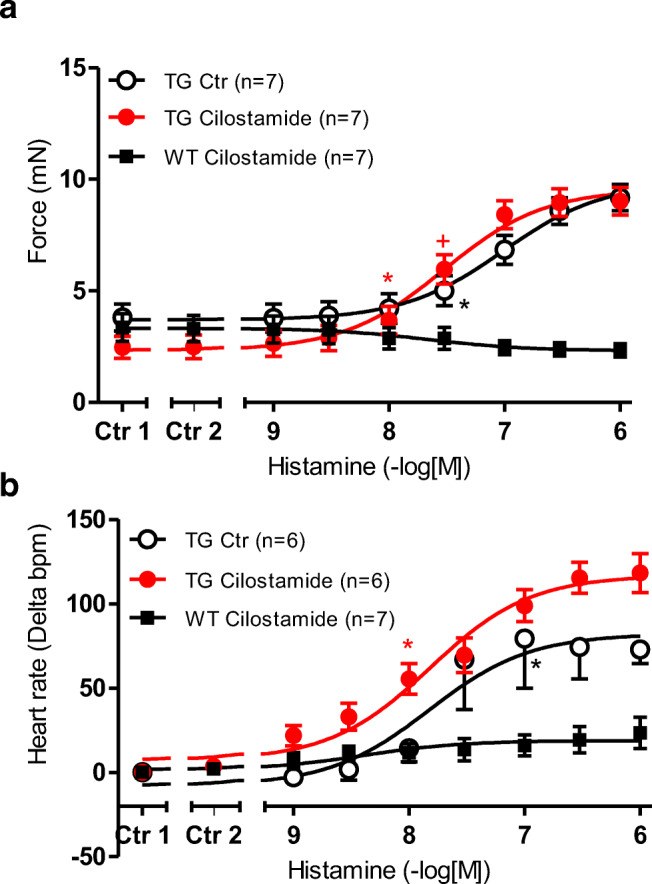
**a** Effect of histamine alone (open circles) or in the additional presence of 1 μM cilostamide on the force of contraction (FOC) of isolated electrically driven (1 Hz) left atrium of H_2_-receptor overexpressing mice (H_2_-TG, closed circles) or littermate mice (WT, squares). FOC before (Ctr 1) and after cilostamide (Ctr 2) are indicated. FOC is given on the ordinate in milli Newton (mN). **b** Effect of histamine alone (open circles) or in the additional presence of 1 μM cilostamide in isolated spontaneously beating right atrium of H_2_-TG (closed circles) or WT (squares). Beating rate in beats per minute (bpm) is depicted on the ordinate. Beating rates before (Ctr 1) and after cilostamide (Ctr 2) are indicated. Abscissae indicate negative decadic logarithm of increasing histamine concentrations Numbers in brackets indicate number of experiments. ^★, **+**^indicate first significant difference (*p* < 0.05) vs. Ctr or WT

Cilostamide (1 μM), a PDE 3 inhibitor, alone was ineffective to increase the FOC in left atrial preparations from WT (squares) or H_2_-TG (closed circles) (Ctr 2 vs. Ctr 1 in Fig. 3a). Similarly, cilostamide did not increase the beating rate in right atrial preparations from WT or H_2_-TG (Ctr 2 vs. Ctr 1), as seen in Fig. 3 b. However, cilostamide shifted the PIE of histamine to lower concentrations of histamine in H_2_-TG (-log EC_50_ Ctr = 7.03 ± 0.08; -logEC_50_ cilostamide: 7.55 ± 0.06; *p* < 0.05) (Fig. 3a). In contrast, cilostamide failed to alter the EC50 of histamine with respect to the PCE (-log EC_50_ Ctr = 7.49 ± 0.11; versus - logEC_50_ cilostamide: 7.88 ± 0.09).

**Fig. 4:**
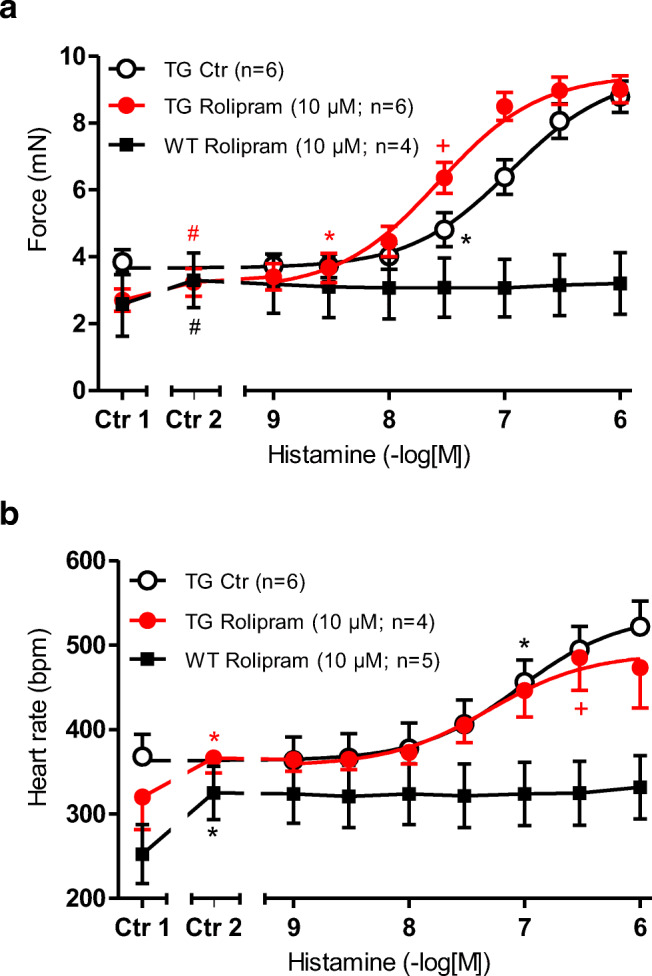
**a** Effect of histamine alone (open circles) or in the additional presence of 10 μM rolipram on the force of contraction (FOC) of isolated electrically driven (1 Hz) left atrium of histamine_2_-receptor overexpressing mice (H_2_-TG, closed circles) or littermate mice (WT, squares). FOC before (Ctr 1) and after rolipram (Ctr 2) is indicated. FOC is given on the ordinate in milli Newton from pre-drug values (mN). **b** Effect of histamine alone (open circles) or in the additional presence of 10 μM rolipram in isolated spontaneously beating right atrium of H_2_-TG atrium (closed circles) or WT (squares). Beating rates before (Ctr 1) and after cilostamide (Ctr 2) are indicated. Abscissae indicate negative decadic logarithm of increasing histamine concentrations Numbers in brackets indicate number of experiments. ^★, **+**^indicate first significant difference (*p* < 0.05) vs. Ctr or WT

Rolipram (10 μM), a PDE 4 inhibitor, increased the FOC in left atrial preparations in WT and in H_2_-TG (Ctr 2 vs. Ctr 1 in Fig. 4a). At the same time T_2_ and dF/dT_max_ amounted to 27.13 ± 1.43 ms and 195.3 ± 74.2 mN/ms in WT and 30.66 ± 2.51 ms and 201.7 ± 23.3 in H_2_-TG under basal, and amounted in the presence of 10 μM rolipram to 26.12 ± 0.89 ms and 250.8 ± 63.5 mN/ms in WT and 28.94 ± 2.67 ms and 258.4 ± 35.2 mN/ms in H_2_-TG (Tables 1 and 2). Rolipram (10 μM) shifted the potency of histamine to increase the FOC (Fig. 4a) (FOC: (-log EC_50_ Ctr = 6.97 ± 0.09; - logEC_50_ rolipram: 7.57 ± 0.06) in H_2_TG and in the presence of 1 μM histamine T_2_ amounted to 26.10 ± 0.74 ms and dF/dT_max_ was measured as 707.4 ± 26.8 mN/ms. Rolipram did not alter the beating rate in WT and H_2_-TG (Fig. 4b). Rolipram did not alter the PCE of histamine in H_2_-TG, but it also did not reveal a PIE of histamine in WT (Fig. 4) or a PCE of histamine in WT (Fig. 4a, b).

### Effects of combinations of PDE inhibitors alone

**Fig. 5:**
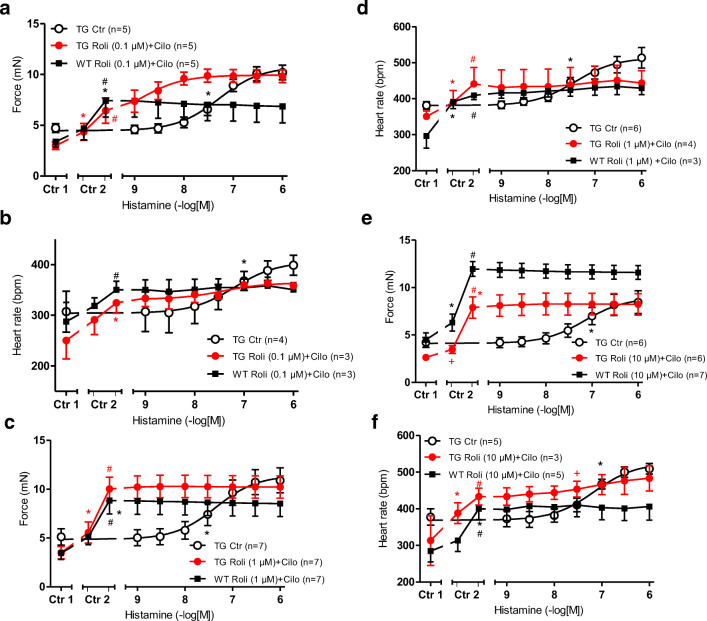
**a** Effect of histamine alone (open circles) in the additional presence of rolipram (0.1 μM) and 1 μM cilostamide on the force of contraction (FOC) of isolated electrically driven (1 Hz) left atrium of histamine_2_-receptor overexpressing mice (H_2_-TG, circles) or littermate mice (WT, squares). FOC before (Ctr 1) and after rolipram (left tick) and cilostamide (right tick, Ctr 2) are indicated. FOC is given on the ordinate in milli Newton (mN). **b** Effect of histamine alone (open circles) in the presence of 0.1 μM rolipram and 1 μM cilostamide in isolated spontaneously beating right atrium of H_2_-TG (circles) or WT (squares). Beating rate in beats per minute (bpm) is depicted before (Ctr 1) and after rolipram and cilostamide (Ctr 2) are indicated on the ordinate. **c** Effect of histamine alone (open circles) or in the presence of 1 μM rolipram and 1 μM cilostamide on the FOC of isolated electrically driven (1 Hz) left atrium of H_2_-TG (circles) or WT (squares). FOC before (Ctr 1) and after rolipram and cilostamide (Ctr 2) are indicated. FOC is given on the ordinate in milli Newton (mN). **d** Effect of histamine alone (open circles) or in the presence of 1 μM rolipram and 1 μM cilostamide in isolated spontaneously beating right atrium of H_2_-TG or WT. Beating rate before (Ctr 1) and after rolipram and cilostamide (Ctr 2) are indicated. Abscissae indicate negative decadic logarithm of increasing histamine concentrations. Numbers in brackets indicate number of experiments. ^★, **+**^indicate first significant difference (*p* < 0.05) vs. Ctr or WT. **e** Effect of histamine alone (open circles) or in the presence of 10 μM rolipram and 1 μM cilostamide on the FOC of isolated electrically driven (1 Hz) left atrium of H_2_-TG (circles) or WT (squares). FOC before (Ctr 1) and after rolipram and cilostamide (Ctr 2) are indicated. FOC is given on the ordinate in milli Newton (mN). **f** Effect of histamine alone (open circles) or in the presence of 10 μM rolipram and 1 μM cilostamide in isolated spontaneously beating right atrium of H_2_-TG or WT. Beating rate before (Ctr 1) and after rolipram and cilostamide (Ctr 2) are indicated. Abscissae indicate negative decadic logarithm of increasing histamine concentrations. Numbers in brackets indicate number of experiments. ^★, **+**^indicate first significant difference (*p* < 0.05) vs. Ctr or WT

The combination of rolipram (0.1 μM) and cilostamide (1 μM) elevated the FOC (Fig. 5) in H_2_-TG (closed circles) and in WT (closed squares, Ctr 2 vs. Ctr 1, Fig. 5a). At the same time T_2_ and dF/dT_max_ amounted to 25.81 ± 1.94 ms and 209.8 ± 38.8 mN/ms in WT and 23.29 ± 1.05 ms and 195.4 ± 27.3 mN/ms in H_2_-TG under basal conditions, and remained unchanged in the presence of 0.1 μM rolipram and 1 μM cilostamide at 25.81 ± 1.40 ms and 513.6 ± 120.9 mN/ms in WT and 23.62 ± 0.92 ms or 445.1 ± 91.2 mN/ms in TG (compare Tables 1 and 2). The combination of 0.1 μM rolipram and 1 μM cilostamide increased the beating rate (Fig. 5b) in H_2_-TG (open circles) and WT (closed squares). Rolipram (0.1 μM) and cilostamide (1 μM) shifted the concentration response curve of histamine on FOC in H_2_-TG to lower concentrations (-log EC_50_ Ctr = 7.31 ± 0.06; - logEC_50_ rolipram+cilostamide: 9.06 ± 0.13 (closed circles, Fig. 5a) and 1 μM histamine prolonged t_2_ to 26.06 ± 0.61 ms and elevated dF/dT_max_ to 704.6 ± 47.7 mN/ms.

In WT, the FOC was not further increased by histamine under these conditions (squares, Fig. 5a). In the right atrium, histamine was ineffective to increase further the already augmented beating rate in H_2_-TG (closed circles) or WT (closed squares, Fig. 5b). Using higher concentrations of rolipram, 1 μM rolipram or 10 μM rolipram increased FOC to such high levels that additionally applied histamine was ineffective to raise FOC further in WT as well as H_2_-TG (Fig. 5c, e). At the same time, dF/dT_max_ (but not t_2_) were changed in the presence of 1 μM rolipram or 10 μM rolipram and 1 μM cilostamide (see Tables 1 and 2). Likewise, 1 μM rolipram or 10 μM rolipram in the presence of cilostamide elevated beating rate in WT and H_2_-TG (Fig. 5d, f). Additionally, applied histamine failed to increase beating rate further (Fig. 5d, f).

**Fig. 6:**
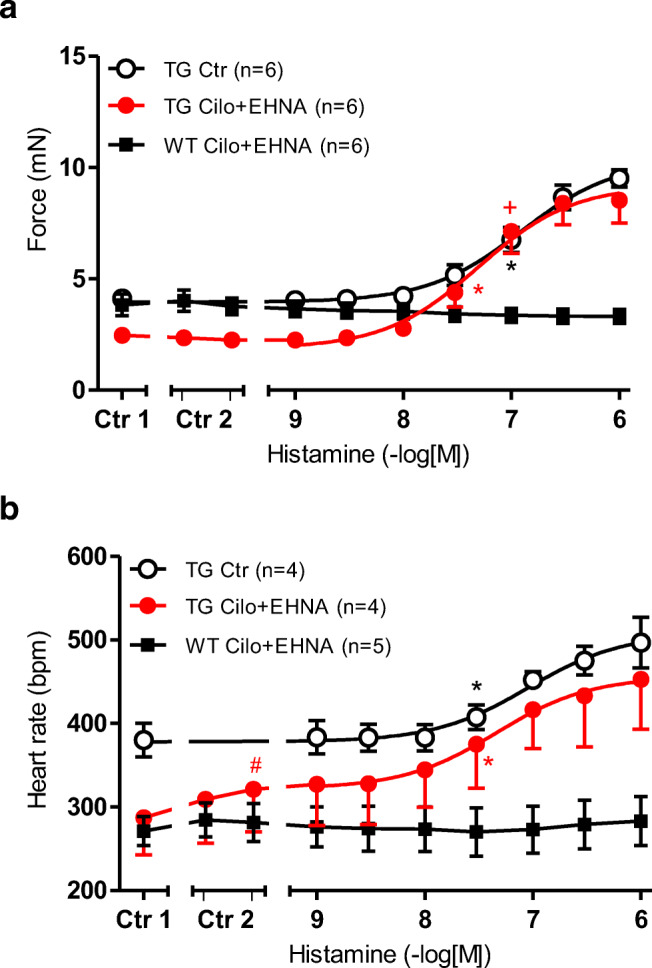
**a** Effect of histamine alone (open circles) or in the additional presence of 1 μM cilostamide and 1 μM EHNA on the force of contraction (FOC) of isolated electrically driven (1 Hz) left atrium of histamine-receptor overexpressing mice (H_2_-TG, closed circles) or littermate mice (WT, squares). FOC before (Ctr 1) and after cilostamide (left tick in Ctr 2) and after EHNA (right tick in Ctr 2) are indicated. FOC is given on the ordinate in milli Newton (mN). **b** Effect of histamine alone (open circles) or in the presence of 1 μM cilostamide and 1 μM EHNA in isolated spontaneously beating right atrium of H_2_-TG (H_2_-TG, closed circles) or WT (squares). Beating rate in beats per minute (bpm) is depicted before (Ctr1) and after cilostamide and EHNA (Ctr2) is indicated on the ordinate

The combination of cilostamide (1 μM) and EHNA (1 μM) did not elevate the FOC (Ctr2 vs. Ctr1 in Fig. 6a) but the beating rate in H_2_-TG (Ctr2 vs. Ctr1 in Fig. 6b). In WT, the FOC and beating rate remained unaltered (Fig. 6a). In the right atrium, under these conditions, histamine did not increase the beating rate in H_2_-TG at lower concentrations (log EC_50_ Ctr = 6.98 ± 0.27; - logEC_50_ cilostamide+EHNA: 7.4 ± 0.24 (closed circles, Fig. 6b).

### Effects of combinations of PDE in the presence of histamine

**Fig. 7:**
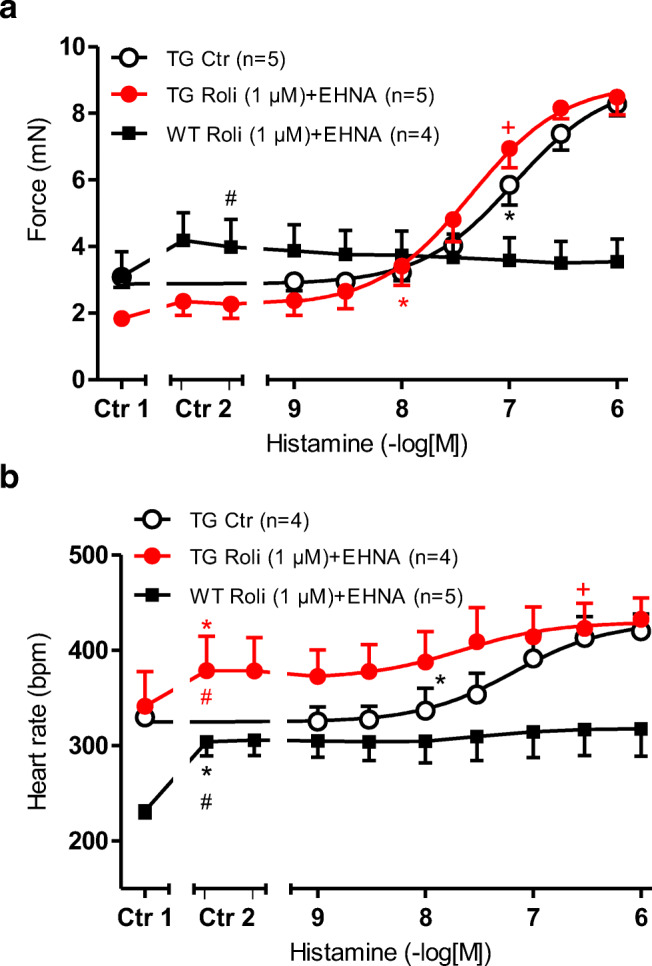
**a** Effect of histamine alone (open circles) or in the additional presence 1 μM rolipram and 1 μM EHNA on the FOC of isolated electrically driven (1 Hz) left atrium of H_2_-TG (closed circles) or WT (squares). FOC before (Ctr1) and after rolipram (left tick), EHNA, (right hand tick in Ctr2) are indicated. FOC is given on the ordinate in milli Newton (mN). **b** Effect of histamine alone (open circles) in the presence of 1 μM rolipram and 1 μM EHNA in isolated spontaneously beating right atrium of H_2_-TG (closed circles) or WT (squares). Beating rate before (Ctr 1) and after rolipram (left tick) and EHNA (right tick, Ctr 2) is indicated on the ordinate. Abscissae indicate negative decadic logarithm of increasing histamine concentrations. Numbers in brackets indicate number of experiments. ^★, **+**^indicate first significant difference (*p* < 0.05) vs. Ctr or WT

The combination of rolipram and EHNA elevated the FOC (Ctr 2 vs. Ctr 1 in Fig. 7a) in WT but hardly in H_2_-TG (Ctr2 vs. Ctr1). At the same time, T_2_ and dF/dT_max_ amounted to 29.10±2.28 ms and 185.0 ± 38.3 mN/ms in WT and 21.47 ± 1.09ms and 115.6 ± 11.6 mN/ms in H_2_-TG under basal, and in the presence of 1 μM rolipram and 1 μM EHNA amounted to 27.06 ± 2.30 ms and 252.9 ± 42.2 mN/ms in WT and 20.87 ± 1.02 ms or 147.5 ± 28.3 mN/ms in H_2_-TG (compare Tables 1 and 2). The combination increased the beating rate in H_2_-TG and WT (Fig. 7b). Under these conditions, the potency of histamine to increase the FOC in H_2_-TG was elevated (log EC_50_ Ctr = 6.89 ± 0.21; - logEC_50_ rolipram+EHNA: 7.43 ± 0.18). Also, the potency of histamine to increase the PCE in H_2_TG was elevated (log EC_50_ Ctr = 7.09 ± 0.22; - logEC_50_ rolipram+EHNA: 7.51 ± 0.31).

**Fig. 8:**
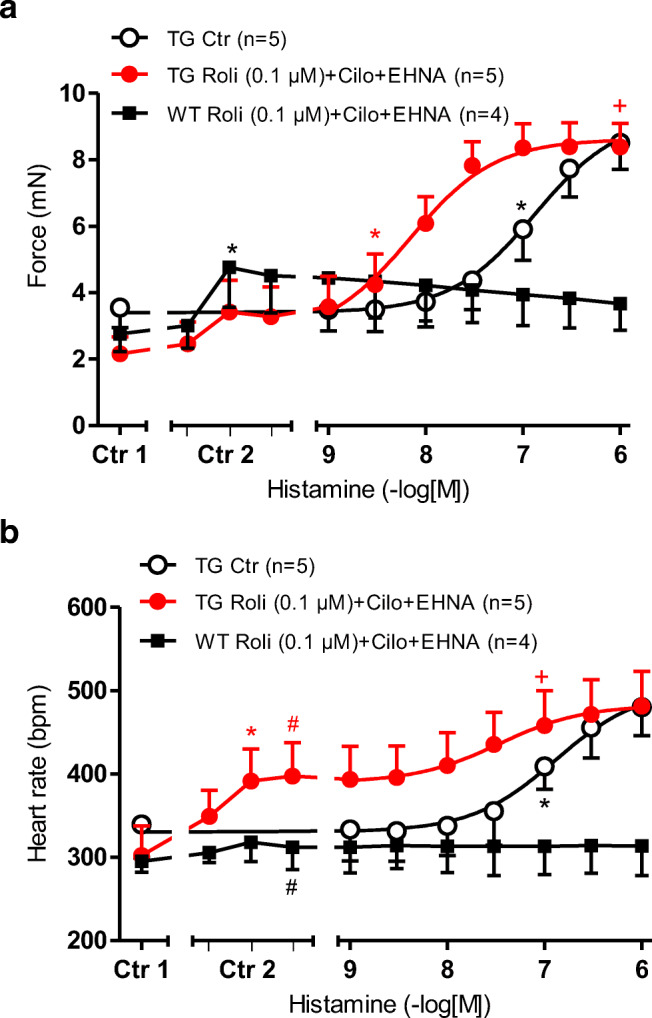
**a** Effect of histamine alone (open circles) or in the additional presence of 0.1 μM rolipram, 1 μM cilostamide and 1 μM EHNA on the FOC of isolated electrically driven (1 Hz) left atrium of H_2_-TG (closed circles) or WT (squares). FOC before (Ctr 1) and after cilostamide (middle tick), EHNA (right tick), and rolipram, (left hand tick in Ctr 2) are indicated. FOC is given on the ordinate in milli Newton (mN). **b** Effect of histamine alone (open circles) or in the additional presence of 0.1 μM rolipram 1 μM cilostamide and 1 μM EHNA in isolated spontaneously beating right atrium of H_2_-TG (closed circles) or WT (squares). Beating rate before (Ctr 1) and after rolipram, cilostamide and EHNA (Ctr 2) is indicated on the ordinate. Abscissae indicate negative decadic logarithm of increasing histamine concentrations. Numbers in brackets indicate number of experiments. ^★, **+**^indicate first significant difference (*p* < 0.05) vs. Ctr or WT

Finally, to inhibit PDE2, 3, and 4 together, we applied a combination of rolipram (0.1 μM), cilostamide (1 μM), and EHNA (1 μM). Under these conditions, the FOC (Fig. 8a) was greatly augmented in WT (Ctr2 vs. Ctr1) and H_2_-TG). At the same time, T_2_ and dF/dT_max_ amounted to 24.78 ± 2.02 ms and 169.3 ± 24.0 mN/ms in WT and 23.70 ± 1.50 ms and 127.5 ± 36.2 mN/ms in H_2_-TG under basal, and were changed in the presence of 0.1 μM rolipram, 1 μM EHNA, and 1 μM cilostamide to 22.66 ± 1.79 ms and 391.5 ± 106.0 mN/ms in WT and 21.66 ± 1.03 ms and 212.4 ± 64.7 mN/ms(compare Tables 1 and 2). Additionally, applied histamine at 1 μM prolonged T_2_ to 24.81 ± 1.06 ms elevated dF/dT_max_ 602.5 ± 52.1 mN/ms(Fig. 8a, Tables 1 and 2).

Moreover, the beating rate was increased by triple inhibition in H_2_-TG (Ctr2 vs. Ctr1 in Fig. 8b) and in WT (Fig. 8b). The response to histamine on FOC and beating rate under triple PDE inhibitions were shifted to the left in H_2_-TG (Fig. 8a, b) (FOC: -log EC_50_ Ctr = 6.89 ± 0.13; - logEC_50_ rolipram+cilostamide+EHNA: 8.36 ± 0.17) (beating rate: -log EC_50_ Ctr = 6.72 ± 0.15; - logEC_50_ rolipram+cilostamide+EHNA: 7.49 ± 0.11, Table 3). No hidden PIE or hidden PCE to histamine was unveiled under these conditions in WT (Fig. 8a, b).

**Table 3:** EC_50_-values of the concentration response curves (CRC) for the PIE of histamine in left atrium of H_2_-TG (top), EC_50_-values of the concentration response curves (CRC) for the PCE of histamine in right atrium of H_2_-TG (bottom), in the presence and absence of the listed PDE-inhibitors. *Cilo*, cilostamide

**EC**_**50**_**-values for the PIE**	Ctr CRC	CRC with PDE inhibition
EHNA	− 7.11 ± 0.11 (*n* = 5)	− 7.01 ± 0.14* (*n* = 5)
Cilo	− 7.03 ± 0.08 (*n* = 7)	− 7.55 ± 0.06* (*n* = 7)
Rolipram	− 6.97 ± 0.09 (*n* = 6)	− 7.57 ± 0.06* (*n* = 6)
Cilo+EHNA	− 6.88 ± 0.17 (*n* = 6)	− 7.27 ± 0.1* (*n* = 6)
Rolipram 0.1 μM+Cilo	− 7.31 ± 0.06 (*n* = 5)	− 9.06 ± 0.13* (*n* = 5)
Rolipram 1 μM +EHNA	− 6.89 ± 0.21 (*n* = 5)	− 7.43 ± 0.19* (*n* = 5)
Rolipram 0.1 μM +Cilo+EHNA	− 6.89 ± 0.13 (*n* = 5)	− 8.36 ± 0.17* (*n* = 5)
**EC**_**50**_**-values for the PCE**	Ctr CRC	CRC with PDE inhibition
EHNA	− 7.13 ± 0.24 (*n* = 3)	− 6.95 ± 0.21 (*n* = 3)
Cilo	− 7.49 ± 0.11 (*n* = 3)	− 7.88 ± 0.09 (*n* = 3)
Rolipram	− 7.11 ± 0.02 (*n* = 4)	− 7.23 ± 0.1 (*n* = 4)
Cilo+EHNA	− 6.98 ± 0.27 (*n* = 3)	− 7.4 ± 0.24 (*n* = 3)
Rolipram 0.1μM+Cilo	− 7.17 ± 0.15 (*n* = 3)	− 7.5 ± 0.03 (*n* = 3)
Rolipram 1 μM +EHNA	− 7.09 ± 0.22 (*n* = 4)	− 7.51 ± 0.31* (*n* = 4)
Rolipram 0.1 μM +Cilo+EHNA	− 6.72 ± 0.15 (*n* = 5)	− 7.49 ± 0.11* (*n* = 5)

*p < 0.05 versus Ctr CRC (control CRC, i.e., in the absence of a PDE-inhibitor)

**Fig. 9:**
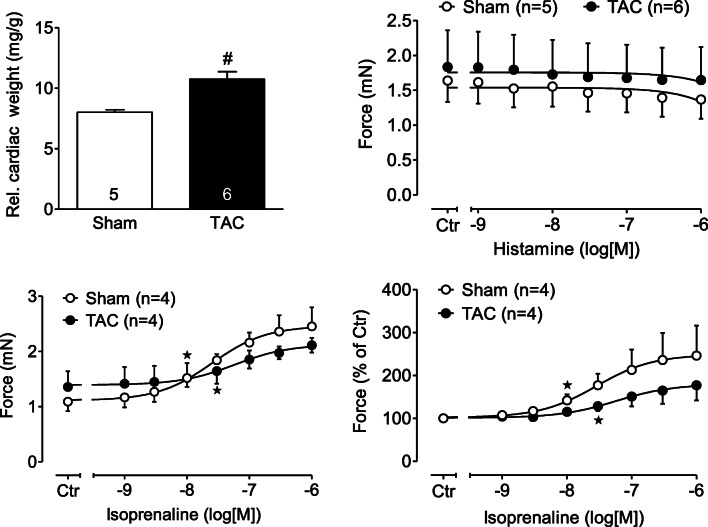
Data on banded mouse hearts Comparison of effects in atrium of H_2_-TG and WT. Relative heart weight was increased upon banding (TAC vs Sham, bar diagram, numbers in bars indicated numbers of mice studied). Histamine failed to increase force of contraction in electrically driven left atrial preparations from Sham- and TAC- mice (upper right inset, ordinate gives developed for in milli Newton (mN). Hypertrophy led to attenuated positive inotropic effects of the β-adrenoceptor agonist isoprenaline. Ordinates in mN (lower left inset) or in percent of pre-drug value (lower right inset). * indicated *p* < 0.05 versus pre-drug values (Ctr). Numbers in brackets indicate the numbers of mice studied

Moreover, we wanted to know whether a hidden PIE to H_2_ could be detected under pathological conditions in WT animals. Hence, we studied the effect of histamine on FOC in left atria from mice with aortic banding and sham operated animals. In mice with aortic banding, the relative hearts weight was higher than hearts from sham operated animals (data not shown). However, histamine (or serotonin studied for comparison) failed to increase FOC (Fig. 9). However, in the same atria, the PIE of the β-adrenoceptor agonist isoprenaline was blunted in atria from banded animals compared to sham operated animals (Fig. 9).

## Discussion

To facilitate the discussion of the present results, we have summarized the main findings in Table 4.

**Table 4:** Synopsis of role of phosphodiesterase (PDE) inhibitors in atrial preparations of WT and H_2_-TG. Increase (↑), decrease (↓) or no alteration (0) in force of contraction (force), beating rate (rate) or changes in EC_50_-values

	Basal force TG	Basal force WT	EC_50_ force TG	Basal heart rate TG	Basal heart rate WT	EC_50_ heart rate TG
PDE2-Inhibition	0	0	**↑**	0	0	0
PDE3-Inhibition	0	0	**↓**	0	0	0
PDE4-Inhibition	**↑**	**↑**	**↓**	0	0	0
PDE2+3-Inhibition	0	0	**↓**	**↑**	0	0
PDE2+4-Inhibition	0	**↑**	**↓**	**↑**	**↑**	**↓**
PDE3+4-Inhibition	**↑**	**↑**	**↓**	**↑**	**↑**	0
PDE2+3+4-Inhibition	0	**↑**	**↓**	**↑**	**↑**	**↓**

**Fig. 10:**
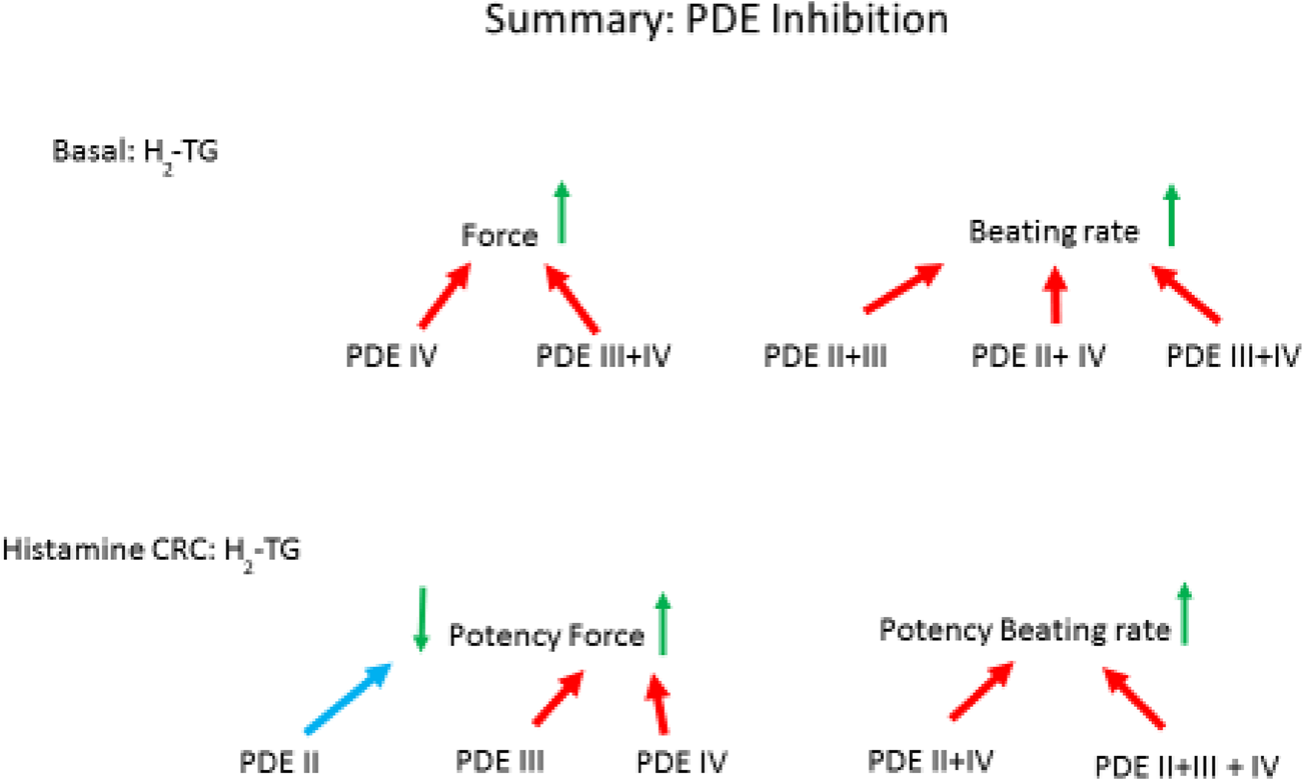
Scheme. Hypothetical simplified explanation which PDE might be mainly involved in the basal (absence of histamine) contractile state in mouse left atrium (Force) or mouse right atrium (beating rate) in H_2_-TG or WT. Likewise the situation if one constructs a concentration response curves to histamine (Histamine CRC) is depicted in the lower half of the figure. This indicated that force under basal conditions can be elevated either by inhibition of PDE IV alone or the joint inhibition of PDEIII and PDE IV In contrast, in the presence of histamine the effect of histamine is potentiated (shifted sinistrally) by either inhibition of PDE III or PDE IV. The interpretation for beating rate is different: only joint inhibition of PDEs will potentiate the chronotropic effect of histamine. In contrast, inhibition of PDE II reduces the potency of histamine to increase FOC in H_2_-TG, conceivably by elevating cAMP and activating PKA in a compartment that can where PDE III can be phosphorylated and activated by PKA

We studied an interaction of PDE inhibition on the PIE and PCE of histamine in H_2_-TG mice, because they mimic the PIE of histamine in the human heart by using the same human H_2_-histamine receptor (Fig. 10). In the future, we would like to extend the present study by investigating the role of PDE isoenzymes for the PIE of histamine in electrically stimulated human atrial strips. We had worked before on the effect of serotonin in the human and mouse heart and noticed similarities to histamine. For instance, we noticed that in WT mice heart, serotonin (5-HT) did not have any effect on force of contraction or beating rate (Läer et al., 1998). This prompted us to generate transgenic mice that overexpress human 5-HT_4_ receptor (which is the receptor subtype responsible for inotropic effect in human atrium and ventricle (e.g., Gergs et al., 2009): we detected an increase in force of contraction by serotonin in 5-HT_4_-TG but no effect in WT (Gergs et al., 2010). For comparison, we started to study histamine, because like serotonin, it can exert an increase in force of contraction in human atrium (via H_2_-histamine receptors) and like serotonin acts in the human heart via an increase in cAMP concentrations and phospholamban phosphorylation. Surprisingly, and like for serotonin, we failed to notice a PIE of histamine in WT atrium (Gergs et al., 2019b, 2020) and therefore, as for serotonin, we generated mice overexpressing human H_2_ receptors and in those mice detected an increase in force of contraction by histamine (Gergs et al., 2019b, 2020, this manuscript). We had reported before that serotonin exerts a positive inotropic effect in rats by action on 5-HT_2_ receptors (Läer et al., 1998). Colleagues in Norway confirmed this work and extended it by showing that in failing rat hearts (due to aortic banding or experimentally induced myocardial infarction) in addition to a 5-HT_2_-receptor mediated PIE also a 5-HT_4_-receptor mediated PIE of serotonin occurred accompanied by increased expression of the mRNA for the 5-HT_4_-receptor (reviewed in: Levy et al., 2008). The positive inotropic effect of 5-HT_4_ receptor stimulation by serotonin in failing rat hearts was potentiated by the PDE inhibitors we used in the current study (Afzal et al., 2008). Based on these rat data, the Oslo group also studied human samples (Afzal et al., 2008) and found that in electrically stimulated human left ventricular trabeculae (from explanted failing heart), PDE 3 and 4 are most relevant for the PIE of serotonin. We used their choice of PDE inhibitors in the past to address the question of the role of specific PDEs in mouse hearts overexpressing 5-HT_4_ receptors (Neumann et al., 2019) and noticed that for the serotonin induced PIE in 5-HT_4_-TG, mainly PDE4 is involved (Neumann et al., 2019). Here, the question arose: can we use the H_2_-TG mouse to find whether and which PDEs decrease the PIE and PCE of histamine in the present mouse model of the human H_2_ receptor? Is the histamine action on force and frequency in H_2_-TG reduced by same endogenous PDEs as that of serotonin in 5-HT_4_ mice or man? Does the effect of histamine on the sinus node of H_2_-TG involve PDE (serotonin acts in sinus node of 5-HT_4_ TG and probably also in living humans without participation of PDEs: Neumann et al., 2019). Using a similar approach, others have studied which PDEs attenuate the PCE of noradrenaline in WT mouse atrium (Galindo-Tovar et al., 2016).

### Right atria, role of PDE isoenzymes in basal conditions

How the heartbeat is generated, maintained, decreased, and increased in the mammalian heart is controversial. Clearly PDEs play a role (for review: Vinogradova and Lakatta, 2009, Kaumann, 2011). In new born pigs, PDE3 and PDE4 controlled the heartbeat while in adult pigs, the PDE4 was mainly important (Galindo-Tovar et al., 2009). Another study detected no PDE2-inhibitor effect on the beating rate in isolated right atrial preparations from WT mice with the compound 100 nM Bay 60-7550 but 1 μM Bay 60-7550 increased the beating rate (Galindo-Tovar et al., 2016). Like the current study, Galindo-Tovar et al. (2016) noted that 10 μM EHNA alone did not increase FOC in isolated WT mouse right atrial preparations. Cilostamide and rolipram, administered together, caused marked increases in sinoatrial rate in isolated right atrium from WT mouse (Galindo-Tovar and Kaumann, 2008).

In our previous work (Gergs et al., 2019b), and in the present work, an increase in the beating rate in WT or H_2_-TG right atria by cilostamide was not detected. Rolipram (1 μM) tended to transiently increase sinoatrial rate in isolated mouse right atrium (Galindo-Tovar and Kaumann, 2008). Inhibition by rolipram and cilostamide together has exerted a substantial PCE (mouse: Galindo-Tovar and Kaumann, 2008, Galindo-Tovar et al., 2009). This is in line with the present findings (Fig. 5b, d, and f).

The addition of cilostamide to a combination of EHNA and rolipram increased the beating rate probably maximally. In healthy humans, the infusion of milrinone, a clinically approved PDE3 inhibitor, led to a sustained increase in the heart rate, suggesting that at least PDE3 is involved in the regulation of the heartbeat in man (Chiu et al., 1999). A new PDE4 inhibitor, namely roflumilast, was reported to have no effect on heart rate in humans (Rabe et al., 2005).

### Right atria, role of PDE isoenzymes with histamine

As in the present study and our earlier study (Gergs et al., 2019b), others noted that the combination of 300 nM cilostamide with 1 μM rolipram increased the beating rate in right atrial preparations of WT mice to a maximum, making it impossible to stimulate the beating rate further with isoprenaline (Galindo-Tovar and Kaumann, 2008).

Galindo-Tovar et al. (2009) argued that the lack of potentiation of the chronotropic effects of rolipram, cilostamide, and concurrent rolipram and cilostamide means that the cAMP pool governing H_2_-receptor-mediated sinoatrial tachycardia is protected from PDE3 and PDE4 and represents a compartment distinct from the cAMP compartment in which both PDE3 and PDE4 reduce basal sinoatrial beating. They speculated that increases in maximum PCE of serotonin in the presence of rolipram and concurrent rolipram plus cilostamide could be due to additivity of the tachycardia caused by 5-HT and the PDE inhibitors (Galindo-Tovar et al., 2009).

### Left atria, role of PDE isoenzymes under basal conditions

Rolipram but not cilostamide alone increased the FOC in isolated left atrial preparations from adult pigs. However, the combination of rolipram and cilostamide increased the FOC significantly (Galindo-Tovar et al., 2009). Cilostamide (300 nM) did not significantly increase left atrial contractility in WT mice (Galindo-Tovar and Kaumann 2008), which agrees with our previous (Gergs et al., 2019b) and the present study’s observations.

### Left atria role of PDE isoenzymes with histamine

In ventricular preparations from pig, rat, and man, serotonin usually fails to induce a PIE. However, this PIE can be disclosed by preincubation with cilostamide alone (in rat and man) and is even higher in the combined presence of cilostamide and rolipram (rat and man: Afzal et al., 2008, pig: Galindo-Tovar et al., 2009). It is noteworthy that in WT mice, neither cilostamide, nor rolipram, nor EHNA, nor their combinations, unveiled a PIE to 5-HT(Gergs et al., 2019b) or to histamine (this study).

### Limitations of the study

One micromolar of EHNA is likely insufficient to inhibit mouse cardiac PDE2 completely. The IC50 value in human heart for PDE 2 inhibition was reported by 0.8 μM (Podzuweit et al., 1995). Looking at Fig. 2 in Podzuweit et al. 1995, one can estimate that 1 μM EHNA can inhibit about 60 % of total PDE2. An advantage is that, at 1 μM, EHNA does not yet inhibit the activity of human cardiac PDE 3 and/or PDE 4 (Podzuweit et al., 1995). In constrast, Méry et al. (1995) found that 10 μM EHNA is needed to inhibit the PDE 2 in the heart sufficiently. However, Méry et al. (1995) studied PDE 2 activity from frog heart and frogs are not a mammalian species, in contrast to mice. Hence, we think that PDE inhibitor EHNA (namely 1 μM) was reasonable choice for the mouse heart in the present work. In subsequent studies, one might use the newer PDE 2 inhibitor Bay 60-7750 instead of EHNA. However, we wanted to facilitate direct comparison to our earlier work on 5-HT_4_ receptors stimulation and PDE2 where we also used 1 μM EHNA (Neumann et al., 2019).

Interestingly, PDE2 inhibition by EHNA can reduce the potency of histamine to increase FOC in left atrium of H_2_-TG (Fig. 3c). This might come about in an indirect way: PDE 3 also hydrolyses cGMP. Hence, if PDE 3 is inhibited, cGMP levels in the heart will rise (Maurice et al., 2014). The generated cGMP can allosterically stimulate PDE 2 activity (Martins et al., 1982; cartoon in Fig. 8b in Neumann et al., 2019). On the other hand, an increase in cAMP would also activate cAMP-dependent protein kinase. Activated cAMP-dependent protein kinase would phosphorylate and activate PDE 3 and PDE 4 (Smith et al., 1991; MacKenzie et al., 2002). Hence, one might envision the following steps: the PDE 2 inhibitor EHNA can increase cellular cAMP (in a certain subcellular compartment relevant for inotropy), this would activate cAMP-dependent protein kinase and subsequently PDE 3 and 4 are activated and might reduce cAMP levels, at least in that compartment that would normally increase force of contraction after H_2_-receptor stimulation in H_2_-TG. In this way, EHNA might reduce the potency of histamine. Moreover, we find that PDE4 is involved in several processes (Table 4). This might be due to the expression of different isoforms of PDE 4 in the heart. For instance, PDE 4 isoforms have been reported to have different subcellular locations in the heart or other tissue (review: Maurice et al., 2014, cartoon: Fig. 1, this work). On the one hand, PDE 4 activity can be increased by activation of cAMP-dependent protein kinases (Maurice et al., 2014). On the other hand, inhibition of PDE 4 would presumably increase cAMP levels. Moreover, an increase in cAMP leading to an increase in the activity of cAMP dependent protein kinase would phosphorylate and activate PDE 3. Thus, rolipram might also increase PDE 3 activity in this indirect way (Maurice et al., 2014). Hence, indirect effects of rolipram might contribute to its contractile effect in the present study, not only its direct effects on PDE 4. Another caveat is in order: even though some of the experiments for beating rate (Table 3, bottom) show no significant effects of PDE inhibitors on EC_50_-values, the number of experiments is quite low in some experimental groups and with a higher number of experiments significant effects of PDE inhibitors might become visible.

In summary, we show for the first time that tachycardia induced by histamine on the human H_2_ receptors is modulated by PDEs only in concert while PDE 3 or 4 alone are sufficient to attenuate the histamine-induced PIE in atrium from H_2_-TG, providing another example for functional compartments of PDE in the mammalian heart.
